# The Brazilian Society of Nephrology Code of Conduct: a bioethical analysis

**DOI:** 10.1590/2175-8239-JBN-2021-0061

**Published:** 2021-07-19

**Authors:** Fábio Humberto Ribeiro Paes Ferraz, Cibele Isaac Saad Rodrigues

**Affiliations:** 1 Fundação de Ensino e Pesquisa em Ciências da Saúde, Escola Superior de Ciências da Saúde, Departamento de Graduação em Medicina, Brasília, DF, Brasil.; 2 Pontifícia Universidade Católica de São Paulo, Faculdade de Ciências Médicas e da Saúde, Departamento de Medicina, Programa Mestrado Profissional em Educação nas Profissões de Saúde, Sorocaba, SP, Brasil.

**Keywords:** Bioethics, Codes of Ethics, Ethics, Medical, Morals, Nephrology, Bioética, Códigos de Ética, Ética Médica, Princípios Morais, Nefrologia

## Abstract

**Introduction:**

Professional deontology can be defined as a set of principles, values and rules of conduct to be applied in the exercise of functions and inherent to a given profession. Nephrology was one of the medical specialties most affected by the mismatch between the accelerated technological development and the ethical dilemmas resulting from it. Recently, the Brazilian Society of Nephrology (SBN) edited its code of conduct, which until then did not exist.

**Method:**

Qualitative study with content analysis of the chapters and articles of the SBN Code of Conduct, from the perspective of principlism bioethics.

**Results:**

The four moral principles of beneficence, non-maleficence, autonomy and justice were found asymmetrically throughout the document, with beneficence predominating over the others.

**Discussion:**

The SBN Code of Conduct predominantly expresses the ethical duties that an associate must comply with, but also restrictions on malfeasance, autonomy and justice, anchoring decision-making by managers and including the distribution of possible punishments. It is an unfinished document; therefore, it must be periodically revised, as expected, due to the rapid technological changes, as well as the need for constructive moderation in the relations of nephrologists with each other and, between them, with the Industry, as well as all the ethical consequences arising from these factors.

## Resumo

**Introdução:**

A deontologia profissional pode ser definida como determinado conjunto de princípios, valores e regras de conduta a serem aplicadas no exercício das funções e inerentes a uma determinada profissão. A nefrologia foi uma das especialidades médicas mais afetadas com o descompasso entre o acelerado desenvolvimento tecnológico e os dilemas éticos decorrentes dele. Recentemente, a Sociedade Brasileira de Nefrologia (SBN) editou seu código de conduta, até então inexistente.

**Método:**

Estudo qualitativo com análise de conteúdo dos capítulos e artigos do Código de Conduta da SBN, sob a ótica da bioética principialista.

**Resultados:**

Os quatro princípios morais de beneficência, não maleficência, autonomia e justiça foram encontrados de maneira assimétrica em todo o documento, com predomínio da beneficência sobre os demais.

**Discussão:**

O Código de Conduta da SBN exprime de maneira predominante os deveres éticos a que um associado deve se atentar, mas também restrições à maleficência, autonomia e justiça, ancorando a tomada de decisão pelos gestores e incluindo a distribuição de possíveis punições. É um documento inacabado, portanto deve ser revisto periodicamente, como previsto, em virtude das rápidas transformações tecnológicas, bem como da necessidade de moderação construtiva das relações dos nefrologistas entre si e, destes, com a Indústria, bem como todos os desdobramentos éticos advindos desses fatores.

## Introduction

Professional deontology is a set of principles, values ​​and rules of conduct inherent to a given profession^1^. The first codes of ethics in the medical field emerged in the 19th century, coinciding with the first liberal medical associations^2^.

Several of these manuals incorporated elements of the Hippocratic Oath, especially related to privacy, beneficence, non-maleficence and paternalism in the doctor-patient relationship^2^.

The emergence of Bioethics led to questions about Cartesian rationalism and the scientific neutrality of physicians and researchers, creating the need for ethical principles that would guide the biomedical activity and the conduct of clinical research^2^^,^ 3.

In this context, principlism theory had a strong influence in the field of bioethics, defending the existence of four universal ethical principles for conflict resolution: beneficence, non-maleficence, autonomy and justice^4^.

Nephrology was one of the medical specialties most affected by ethical dilemmas resulting from the accelerated scientific-technological development in the post-war period, with nephrologists often exposed to difficult ethical decisions, especially in a context of scarcity of resources^5^^,^ 6. It is no coincidence that most Nephrology Societies were created during this period, such as the Brazilian Society of Nephrology (SBN), founded in 1960^7^.

The edition of the SBN Code of Conduct took place only in the year 2020^8^, having as one of the main objectives to fill this gap.

Thus, this study aims to analyze the bioethical content of the SBN Code of Conduct, aiming to promote an understanding of the ethical issues included in it.

## Methods

This is a qualitative study carried out through content analysis of pre-selected bioethical literature.

**Figure 1:**
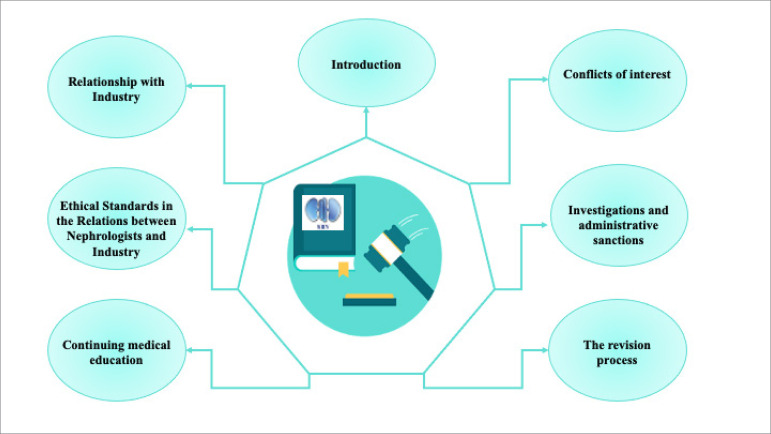
Thematic chapters present in the 2020 Code of Conduct of the Brazilian Society of Nephrology.

As a documentary sample, we selected the SBN Code of Conduct^8^, a document consisting of 6 pages in which there are 30 articles in 7 chapters, as shown in Figure 1.

The material exploration phase consisted of floating reading, followed by careful and exhaustive analysis of the entire document, aiming to give meaning to each message.

In MS-Word software tables, all content was transcribed in full, with identification and categorization of its main bioethical content, having as reference the principles of principlism bioethics, already described^4^.

In order to standardize the understanding of each principle, we adopted the following definitions^3^:

Beneficence: the duty - that is, a morally active attitude - to do good;

Non-maleficence: need to avoid/mitigate damage;

Autonomy: ability to act freely, consciously and deliberately with specialized knowledge and without external coercion;

Justice: impartiality in the distribution of risks and benefits, with equal treatment for equal entities and unequal treatment for unequal entities.

In situations where the concepts of non-maleficence and autonomy (mainly related to the restriction of autonomy of nephrologists) were found to be superimposed, it was decided to understand the first as related to institutions (the SBN itself) and the second as related to individuals (SBN members).

## Results

In the systematized and categorized content analysis of the SBN Code of Conduct, we found the presence of all principlism bioethics principles distributed asymmetrically in the different chapters; with a predominance of the principle of beneficence over the others (see Table 1).

**Table 1:** Princípios bioéticos presentes nos capítulos do Código de Conduta da SBN

	Beneficence	No Maleficence	Autonomy	Justice
Introduction				x
Relationship between SBN and the Industry	x	x	x	
Continuing Medical Education	x		x	x
Conflict of Interest	x	x	x	
Ethical Standards between Nephrologists and the Industry	x	x	x	
Ex-officio assessment	**x**	**x**		x
Preparing the Review	x			

Below is the description of the principles according to each corresponding chapter:

### 1. Introduction

*Justice*: The Code of Conduct is intended for all members without distinction, and its violation is subject to administrative sanctions. All partners are interpreted as equal, and the distribution of possible losses must be equal.

### 2. Relationship between sbn and the industry

*Charity*: Explains that SBN's mission is to promote the growth of the specialty (Article 1), and that research developed by the industry must be conducted in an ethical, transparent manner, and in accordance with the current legislation (Article 2), with these two issues being moral duties to be met.

*Non-maleficence*: The SBN may enter into partnerships for educational and scientific programs through agreements or contracts (Article 3); there is a prohibition on the commercial promotion of companies without a mutual agreement with the executive board, except in defined spaces (article 4). In this way, SBN would be in harm's way if it did not restrict the performance of companies.

### 3. Medical and continuing education

*Charity*: The studies developed by the members must be based on evidence, use scientific methodology and observe ethical principles (Article 6), with the duty to report fraud and unethical conduct being a moral obligation (Article 7).

*Autonomy*: Brazilian speakers must be active and compliant (Article 8) and may receive fees at educational events sponsored by the industry that are reasonable to those stipulated in the market (Article 10), but must only address topics of education, training and/or the correct use of the company's own products (Article 11). Thus, there are restrictions on the member's autonomy over his area of ​​expertise.

### 4. Conflicts of interest

*Charity*: SBN members must be aware of institutional commitments (Article 12), and any conflict of interest must be declared (Article 13).

*Autonomy*: Nephrologists who hold paid positions in the industry and/or have employment relationships with them are not eligible to run for positions in the National and Regional Board of Directors (Article 14), and cannot express opinions on behalf of the SBN without specific delegation (Article 15). Again, there is a restriction of autonomy to the performance of the partners.

*Non-maleficence*: SBN associates may not use positions within the SBN to obtain advantages for themselves or for companies (Article 17). More than a restriction on autonomy, there is a clear guidance on avoiding/mitigating the occurrence of damage.

### 5. Ethical standards in relations between nephrologists and the industry

*Charity*: Prescription of drugs and treatments must be based on scientific evidence (Article 18), and any conflict of interest must be resolved considering the health, interests and well-being of the patient (Article 19). The SBN associate acting as an investigator in an industry research project must comply with relevant legislation and ethical recommendations of good practice (Article 21). Again, the verb "ought" implies an ethical obligation to be fulfilled.

*Autonomy*: Members must not accept financial incentives from the industry for the use of equipment, supplies and medicines (Article 20). Again, there is a restriction on the individual autonomy of the partners.

*Non-maleficence*: The President, the members of the National Board of Directors and all associates may not provide or contract, directly or indirectly, products or services paid for by the SBN (Article 23). The malfeasance would be for the institution to act on its own to the detriment of its partners. In this context, a better specification of which acts would be formally discouraged could give greater clarity to that paragraph.

### 6. Ex-officio assessment or administrative sanctions

*Charity*: Our Legal Department and the National Board must be notified in the event of a Code violation (Article 24), and the President of the SBN must initiate an investigation process if there are indications of such breach (Article 27). These articles reinforce the moral duty to enforce the code of conduct.

*Justice*: Secrecy and confidentiality will be guaranteed to informants and defendants (article 25), and the verification process will guarantee constitutional, adversarial rights and full defense (article 29). Thus, there is equitable, fair and equal treatment between plaintiffs and defendants.

*Non-maleficence*: In the absence of evidence of infringement, the commission will dismiss the case (Article 28). Failure by the commission to dismiss it would imply malfeasance by the member, when misconduct is not proven.

### 7. Preparation of the review

*Beneficence*: The Code of Conduct must be revised every 4 years (Article 30). In this way, it is constant updating in case new ethical paradigms generating good for all involved.

## Discussion

The content analysis of the SBN Code of Conduct shows the influence of principlism bioethics in its writing, with the identification of the principles of beneficence, non-maleficence, autonomy and justice distributed asymmetrically throughout the document, with a predominance of beneficence over the others.

One of the possible interpretations is that the wording of the document had as one of its objectives to be a "moral compass" on the ethical duties and imperatives that such members should follow, and not just a document that aimed to restrict autonomy and distribute punishments fairly and equitably.

We checked four chapters of the aforementioned code of conduct (which deal with the relationship between SBN and the industry, medical and continuing education, conflict of interest and ethical standards in the relationship between nephrologists and the industry) with the current medical literature, which addressed issues such as: quantifying the magnitude of incentives provided by the industry^9^, identifying the impact and effect of such incentives on drug prescription^10^ and recognizing the existence of a conflict of interest, not only among physicians, but among Medical Associations themselves, that represent them and the industries that finance scientific and academic events^11^.

The first edition of the SBN Code of Conduct is undoubtedly a great advance, but, like most contemporary codes of ethics, it does not address all ethical and moral dilemmas. It should be noted that it is an unfinished document, which must be periodically revised due to rapid technological changes, as well as because of the need for constructive moderation in the relations of nephrologists among themselves and, between them and the Industry, in addition to the ethical consequences arising from these issues.
